# Integrated survey of *Taenia solium* endemicity, schistosomiasis, scabies, and Guinea-worm disease in northern Namibia

**DOI:** 10.1186/s40249-026-01514-w

**Published:** 2026-11-03

**Authors:** Stark Katokele, Undjee Kaura, Sirak Hailu, Wilma Florenze Soroses, Rosalia Nairenge, Elizabeth Juma, Bernadette Abela, Amadou Garba Djirmay, Helena Meke Shikwambi, Maria Amaambo, Nicole Wangui, Nyamai Mutono, Katherine Gass, Meritxell Donadeu, Pauline Mwinzi

**Affiliations:** 1 Ministry of Health and Social Services of Namibia, Windhoek, Namibia; 2 World Health Organization Country Office, Namibia, Windhoek, Namibia; 3 Expanded Special Project for Elimination of Neglected Tropical Diseases, World Health Organization Regional Office for Africa, Brazzaville, Republic of Congo; 4 Global Malaria and Neglected Tropical Diseases Programme, World Health Organization, Geneva, Switzerland; 5 Namibia Institute of Pathology (NIP), Windhoek, Namibia; 6 Center for Epidemiological Modelling and Analysis (CEMA), University of Nairobi, Nairobi, Kenya; 7 Paul G. Allen School for Global Health, Washington State University, Pullman, WA USA; 8 Task Force for Global Health, Decatur, GA USA; 9 Veterinary Biosciences, Melbourne Veterinary School, Faculty of Science, University of Melbourne, Melbourne, VIC Australia

**Keywords:** *Taenia solium*, Neurocysticercosis, Schistosomiasis, Soil-transmitted helminthiasis, Scabies, Dracunculiasis, Guinea-worm disease, Namibia

## Abstract

**Background:**

During a Mass drug administration (MDA) campaign for schistosomiasis using praziquantel in Namibia in 2019, three cases of serious neurological adverse events were observed due to neurocysticercosis caused by the parasite *Taenia solium*. To have a better understanding of *T. solium* endemicity, and to prevent and minimize adverse events in future disease control campaigns, the Ministry of Health and Social Services of Namibia decided to conduct field surveys, and take the opportunity to integrate schistosomiasis, scabies, and Guinea-worm disease (GWD) surveillance.

**Methods:**

In July 2024, ten survey teams were deployed to the five regions selected as high-risk (Kavango West, Ohangwena, Omusati, Oshana and Oshikoto), using the World Health Organization (WHO) Risk Classification tool for *T. solium*. Stool and urine samples were collected and analyzed using Kato–Katz and urine filtration, interviews were undertaken relating to GWD and clinical assessments conducted for scabies. Geographical variations in parasite distribution were evaluated for each species using the design-adjusted Rao-Scott second-order chi-square test of independence.

**Results:**

Roaming pigs and deficient sanitation were confirmed in all regions (61% of the households reported not having a latrine). Laboratory analysis of 4880 stool samples confirmed the presence of *T. solium* in all regions except Oshana. Only Ohangwena, with a positivity rate of 0.6%, reached the threshold suggested by the WHO to trigger a public health program. However, *T. solium* was also present in another three regions below the WHO threshold, and while it was not detected in Oshana, it was detected in nearby villages in Ohangwena and Oshikoto. The analysis of 4876 urine samples showed the prevalence of schistosomiasis was only above the threshold to trigger MDA in Kavango West (9.98% for *Schistosoma haematobium*, and 1.65% for *S. mansoni*). The overall prevalence of scabies was 1.9% and below the suggested level to justify MDA in all regions. Three households in Ohangwena, near the border with Angola, reported having seen GWD in humans or dogs.

**Conclusions:**

The results provide updated information on *T. solium* and schistosomiasis endemicity and will guide future disease control efforts, including the prevention, early identification and management of praziquantel-related adverse events.

**Supplementary Information:**

The online version contains supplementary material available at https://doi.org/10.1186/s40249-026-01514-w.

## Background

Namibia is a South-west African country with a population of about three million. It is classified as an upper-middle-income country by the World Bank [1] although inequalities persist and Neglected Tropical Diseases (NTDs), usually prevalent in impoverished communities, are still present [2]. Among the parasitic NTDs, schistosomiasis and soil-transmitted helminths are known to be present in Namibia [3, 4]. The World Health Organization (WHO) recommends preventive chemotherapy as the core intervention against those diseases, and the most used modality is Mass drug administration (MDA) in which the eligible population of an area is periodically given an appropriate anthelmintic as a public health tool [5].

The Ministry of Health and Social Services of Namibia (MoHSS) adopted MDA to control schistosomiasis and soil-transmitted helminths, with the first MDA campaign using praziquantel and albendazole conducted in 2015. A second MDA campaign also using praziquantel and albendazole was undertaken in 2019 among school age children in six regions, namely Zambezi, Kavango East, Kavango West, Ohangwena, Omusati and Otjozondjupa. In 2019, at least 158,614 children were dewormed with praziquantel (40 mg/kg) and 169,189 with albendazole [6].

There were three cases of serious adverse events reported from Ohangwena region during the 2019 MDA campaign. Three children aged nine, ten and seventeen years developed serious symptoms the same day the anthelmintics were administered, and all were hospitalized. The nine and ten-year-old girls presented sudden loss of consciousness while a seventeen-year-old boy manifested generalized tonic–clonic seizures. Brain computed tomography scans showed diffuse neurocysticercosis (NCC) in the two girls while the imaging was normal in the seventeen-year-old boy. The nine-year-old girl and the seventeen-year-old boy recovered fully while the ten-year-old girl died after a week of hospitalization. The autopsy confirmed that the deceased had NCC as well as cerebral oedema likely related to adverse reactions from the medications on the *Taenia solium* cysticerci [7]. Since the events of 2019, the MoHSS suspended MDA for schistosomiasis until the distribution of *T. solium* was determined.

Neurocysticercosis is caused by the larval cysts of the parasite *T. solium* present in the central nervous system. The infection can be asymptomatic or result in serious disease and death. While NCC can produce diverse neurological symptoms, the most common clinical manifestations are seizures, epilepsy and intra-cranial hypertension [8, 9]. Taeniasis and cysticercosis caused by *T. solium* are considered as NTDs by the WHO, and the parasite is endemic in many countries including in parts of Namibia [10]. Neurocysticercosis may be treated with anthelminthics in combination with anti-inflammatory medicines under the supervision of a specialist neurologist, but only after imaging has been used to assess the location, number and stage of the parasites, and inflammatory reactions [11]. When praziquantel is used for NCC, it is used at 50 mg/kg per day for 10 to 14 consecutive days, which is a much higher dose than the single dose of 10 mg/kg used for taeniasis, or the single dose of 40 mg/kg used for schistosomiasis [11].

Although praziquantel at the doses used for MDA for schistosomiasis or taeniasis is usually safe, in *T. solium* endemic areas, where there are likely to be people with latent NCC [12] it is possible, that treatment with praziquantel could lead to inflammation around the *T. solium* cysts in the central nervous system, triggering or aggravating neurological symptoms despite the drug being used at much lower doses than those used therapeutically for NCC [13]. This may have been the case for two of the serious adverse events observed during the 2019 MDA campaign, as NCC has been reported in northern Namibia [14]. Similar adverse events have also been observed in other countries such as Madagascar [15].

Limited information is available about the endemicity of *T. solium* in Namibia. A study conducted in the Intermediate Hospital Oshakati reported that 51.4% of the people presenting first time seizures had NCC [14], and many of the risk factors (deficient sanitation and roaming pigs) are present in the area [16]; 71% of the population in rural areas were reported to practice open defecation [17] and indigenous pigs are present in the northern communal areas of Namibia, where they are an important source of nutrition and cash for the communal farmers [18].

The team investigating the serious adverse events of 2019 in Namibia after the schistosomiasis MDA, made key recommendations including conducting a baseline survey to determine the endemicity of *T. solium* in Namibia. A better understanding of where *T. solium* is present is important to develop appropriate control programs and promote good case management, mitigating the risk of adverse events for programs using praziquantel.

The MoHSS decided to conduct a risk assessment to identify endemic and high-risk areas for *T. solium*, followed by a field survey to confirm its endemicity. The surveys were designed to incorporate other priority underfunded public health activities identified by the program.

*Schistosoma mansoni* and *S. haematobium* are known to be endemic in northern Namibia [3]. Cases of scabies had been reported from all the regions of Namibia over the years, and in 2015 the MoHSS declared a scabies outbreak in the entire country [19]. The MoHSS took the opportunity of conducting the field survey for *T. solium* in remote areas, to include surveys for schistosomiasis and scabies. Namibia has never been known to be endemic for Guinea-worm disease (GWD), also known as dracunculiasis. However, Angola reported three human cases in Cunene province, bordering Namibia between 2018 and 2020 [20], 85 infected dogs in 2023, and 36 during January–June 2024 [21]. The MoHSS wanted to make enquiries concerning the possible presence of GWD in northern Namibia.

The main goal of the surveys was to determine the endemicity of *T. solium* in the five areas identified as high and moderate risk using the WHO Risk Classification Tool [22], focusing on the schistosomiasis endemic regions. Each region had its own survey, and they should be seen as five different surveys conducted simultaneously.

The mapping survey had five objectives: (1) To determine if the targeted high and moderate risk areas have *T. solium* taeniasis in humans and if the prevalence is above the threshold of 0.5% for public health intervention [23], (2) to obtain household data on access to sanitation and hygiene, free roaming pigs and consumption of raw/under cooked pork for programmatic decision-making, (3) to estimate the current prevalence and intensity of schistosomiasis in the targeted study areas, (4) to determine the prevalence of scabies among the study communities, and (5) to verify any rumours/information on occurrence of human and/or animal cases of GWD.

## Methods

The methodology involved two steps. The first step was the identification of high-risk areas for *T. solium* through a desk review. The second step involved the field survey and data collection in the selected high-risk areas.

### Identification of *T. solium* endemic and high-risk areas

Risk assessment to identify endemic and high-risk areas for *T. solium* at sub-district level was conducted in 10 regions during the last quarter of 2023 using the WHO mapping protocol and the WHO Risk Classification tool [22]. The areas assessed were known to be endemic for schistosomiasis, namely, Zambezi, Kavango East, Kavango West, Ohangwena, Oshikoto, Oshana, Omusati, Kunene, Otjozondjupa and Omaheke Regions.

The WHO Risk Classification tool was populated with information from the literature, taeniasis information collected from the MoHSS (DHIS2) on suspected and confirmed taeniasis cases, and village level information on the two key risk factors, i.e. open defecation and roaming pigs. The village information was collected in a qualitative manner by the Community Health Workers (CHWs) assigned to the health facilities in the selected regions using a simple rapid assessment tool designed for this purpose (Supplementary file 1). Data on the pig population and porcine cysticercosis was not readily available.

**Fig. 1:**
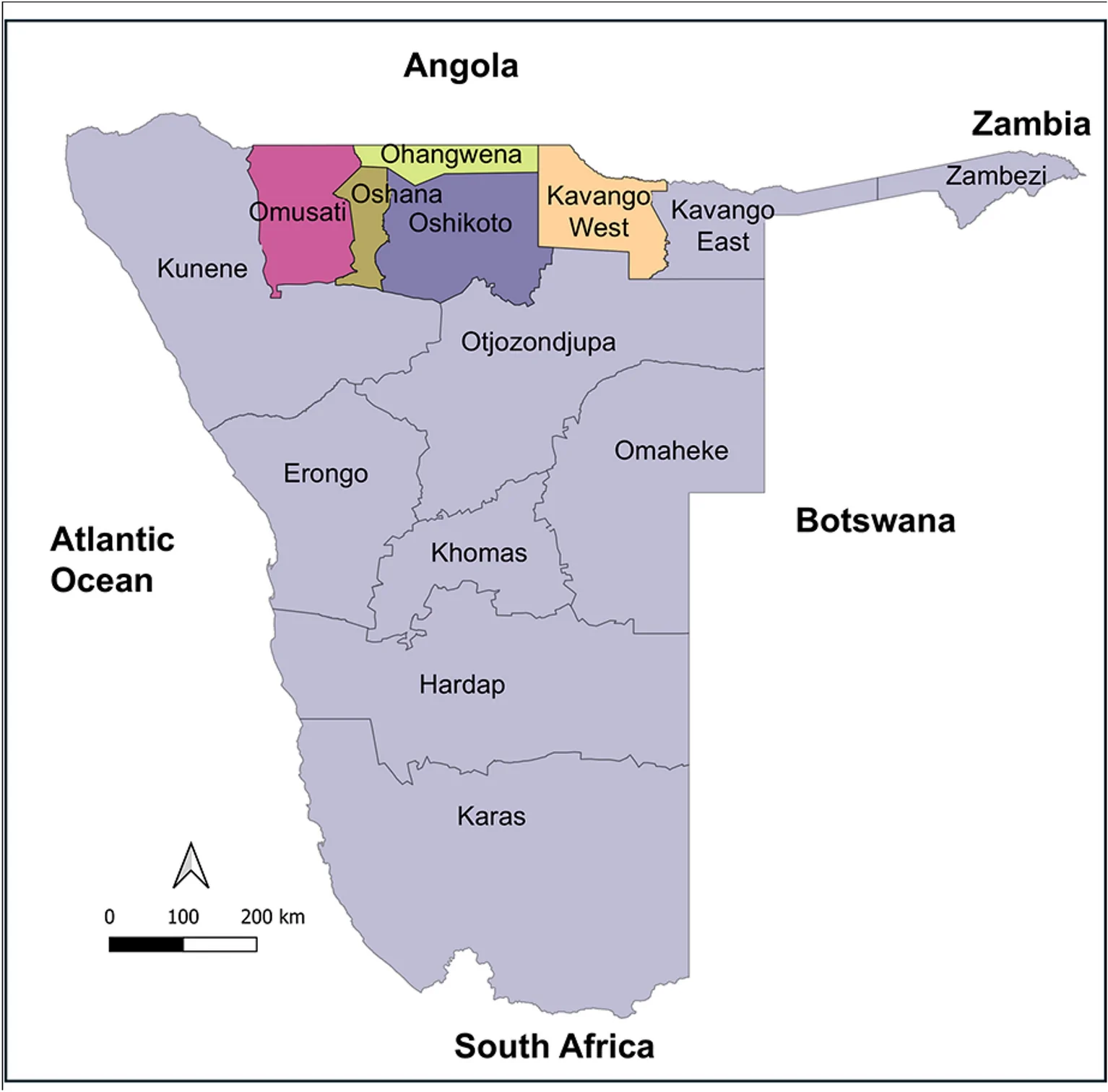
Map of Namibia showing the five regions selected for the field surveys. Map generated using QGIS 3.44.0 (QGIS Development team in https://www.qgis.org/). The map boundary data was obtained from the Humanitarian Data Exchange (HDX) managed by the United Nations Office for the Coordination of Humanitarian Affairs (OCHA) and made available under a Creative Commons Attribution 4.0 (https://data.humdata.org/dataset/cod-ab-nam)

Of the 10 regions assessed, the five with the highest reported number of villages with the coexistence of roaming pigs and deficient sanitation were selected for the field surveys to confirm *T. solium* endemicity (Fig. 1) as described under survey methodology below.

### Survey methodology

#### Study design

The surveys were carried out according to the requirements for conducting a *T. solium* survey as outlined in the WHO *T. solium* monitoring and evaluation framework [24]. The framework had been shared as an advanced draft document by WHO, to take the opportunity to validate it, and receive feedback on clarifications needed or any missing aspects. The methodology used for selection of the survey area, sample size, village selection, number of samples per village and sampling was as detailed in the final published document [24].

#### Sample size

In short, the sample size per sampling unit was chosen to determine if a survey area had taeniasis above a threshold of 0.5% [23, 24], which would trigger a public health intervention. According to the WHO monitoring and evaluation framework, a sample size of 960 was required from each of the five regions. An extra 20% was included to account for non-response, absenteeism or other causes of loss, resulting in 1200 individuals targeted in each sampling unit (region).

Purposive sampling was used to target high-risk communities so only communities with the key risk factors, that is, roaming pigs and deficient sanitation were sampled [24]. The sampling units comprised a region consisting of health facility catchment areas and villages or communities. For each sampling unit, two-stage cluster random sampling was employed. In the first stage, 20 villages (clusters) were selected randomly using the WHO Excel tool for village selection [25] based on a list of all the at-risk villages in the region. The original target was to obtain 48 samples per village (960/20 = 48), but considering the extra 20%, the field target was 60 (1200/20 = 60) samples per village. As two samples were to be collected per household, in the second stage, 30 households (60 samples/2 samples per household = 30 households) were targeted within each cluster/village.

In other words, two individuals were to provide samples from each household, aiming at 30 households per selected village (20 villages were selected per region), which would mean 600 households per region, with the objective to get at least 24 households per village, which would equal 480 households per region.

#### Sensitization and participant selection

A team of representatives from the MoHSS and WHO travelled to all the 5 regions to conduct regional sensitization meetings and community mobilization. Their activities involved meetings with the regional and district health management teams, the regional governors, councilors, and community leaders, as well as with health program officers and local health workers. Radio interviews on the Namibian Broadcasting Corporation (NBC) were conducted in the local languages. Church announcements, materials in local languages, and discussion groups were also used for community mobilization.

In the absence of a complete list of households at village/cluster level, a “*Random Walk approach*” was planned to select the households as described in Box A2.3 of the WHO monitoring and evaluation framework for *T. solium* [24]. However, due to the difficult terrain and the vastness between the households, the initially planned random selection of the households was changed for the snowball sampling technique, in which identified participants nominate future participants through community health workers or community volunteers who identified the households in the villages.

Two consenting/assenting family members aged 5 years and above were selected randomly (the head of the household was requested to volunteer the individuals) to provide stool and urine samples for parasitological analysis. It was important to have a mix of adults and children for the stool and urine samples to ensure that all the people who consumed pork were represented. Most households had children and adults, but in the absence of children, the next house was selected. For scabies, all consenting adults and assenting children were clinically assessed.

#### Survey teams and data collection

During July 2024, ten survey teams (two per district), were organized by the MoHSS to conduct the field work. Each survey team comprised two nurses (one conducted the clinical assessment of scabies, GWD investigations and acted as data recorder, and the other acted as biological sample collector), a CHW, and a driver. The survey team members were trained by a team composed of MoHSS and WHO staff and consultants for five days, including practical exercises during the last two days of the team training.

Survey teams moved from house-to-house and were supported by a local guide wherever feasible. The survey teams were expected to survey an average of 15 households per day due to the difficult terrain and sparse settlements of the population. Two teams were allocated for each sampling unit (region) to complete the 30 households in a village/cluster per day and the 20 clusters in the region within 25 working days.

*Household Census and Laboratory Sample Collection Form*: Individual data including name, age in years at most recent birthday and gender were recorded for all family members in the household using the *Household Census and Laboratory Sample Collection Form*. Absences, refusals, and the laboratory samples collected were also recorded in this form.

#### Coordination, supervision and additional support

Five trained supervisors (one per region) were assigned to oversee the survey implementation and to support the two teams in their respective regions. An overall survey coordinator was responsible for managing the field work. Weekly meetings were held by the Central Coordination team composed of the survey coordinator, WHO support team, regional supervisors, Information Technology, Liaison officer, administration and the MoHSS research unit, to ensure adherence to the protocol and compliance with research ethics.

#### Collection of biological samples

Two persons per household were each given two 100 ml sample collection containers for stool and urine on the previous day or on the same day as sample collection. The participants were instructed to introduce a small quantity (10 g) of stool using a wooden stick/spatula, tightly close the container without contaminating its outside, and to maintain strict personal hygiene [24]. The stool and urine samples collected were given unique identification number and recorded on the Household Census and Laboratory Sample Collection Form.

The samples were packaged and labeled in the field, kept in the cool box with ice packs immediately after collection to preserve their integrity and then sent to the nearest Namibia Institute of Pathology (NIP) laboratory for appropriate storage and analysis.

#### Household and WASH (water, sanitation and hygiene) questionnaire

The household head or their proxy was asked questions relating to:

- Household: information related to the household head, number of household members, presence of roaming pigs, and pork eating habits.
- WASH: source of drinking water and place of defecation. This was followed by an observation of the latrine, handwashing facilities, and availability of water.

#### Guinea-worm search

At the end of the scabies clinical assessment (detailed below), the nurse showed the picture of GWD (human and animal cases—Supplementary file 2) and asked if the person had seen this disease in this or any other community during the past 12 months. If the answer was YES, the interviewer followed up with additional questions to identify the exact location and details.

### Diagnostics

*Taeniasis, schistosomiasis and other helminths:* Besides *Taenia* spp. and *Schistosoma* spp., other helminths assessed were hookworms, *Ascaris lumbricoides, Trichuris trichiura, Enterobius vermicularis, Fasciola* spp.*, Hymenolepis nana* and *Diphyllobothrium latum*. The methodologies used were the Kato–Katz and urine filtration as described in the WHO Bench aids manual [26]. Ideally these techniques use reading of two slides per sample to increase the sensitivity of the test, however, due to resource constraints, only one slide was analysed, and as quality control, every 10th slide was read by two experienced laboratory technicians. A refresher course was conducted for the laboratory personnel on the Kato–Katz and urine filtration techniques, especially for identification of *T. solium* eggs in the Kato–Katz. In addition, two experienced medical scientists from Madagascar and Tanzania spent 10 days with the laboratory technicians to ensure quality was maintained.

Taenia *species confirmation*: Because Kato–Katz does not allow *Taenia* species differentiation, Kato–Katz positive samples were preserved in ethanol (2–5 g of stool stored in at least double the volume of ethanol 95%) and sent to the University of Melbourne in Australia for species confirmation using molecular methods. In short, DNA was extracted from the egg-positive samples using a commercial kit, a rrnS PCR was conducted followed by sequencing as described by Lightowlers et al. [27].

*Scabies clinical assessment:* For each participant, a standardized examination for the detection of scabies was conducted. The team collected data on the presence of itch and on reported close contacts having an itch or rash. In individuals over 1 year of age, scabies examination was limited to easily exposed body areas such as foot, leg to the thigh, hand to the upper arm, scalp, and neck since a full body examination was not practical in the field. This approach has been validated to have a sensitivity of >90% for the detection of scabies [28]. However, if an adult reported a lesion elsewhere and requested it to be examined, this was done privately. Due to a wider distribution of lesions in infants, a full skin examination was conducted for those below 1 year of age. All sensitive examinations were performed privately and in the case of children, with the presence of a guardian.

The nurses wore disposable examination gloves during scabies clinical assessments and recorded the presence of scabies lesions (typical or atypical), and recorded whether there was itching, or signs of superinfections. To avoid misdiagnosis, if there were any doubts, with the permission or assent of caregivers, the nurses took a photo of the affected skin to discuss with the supervisor or a dermatologist.

Cases were classified using the 2020 International Alliance for the Control of Scabies (IACS) consensus criteria [29]. Cases were classified as positive for scabies if they fulfilled either the criteria for clinical scabies or suspected scabies based on lesion appearance, lesion distribution, history of itch and contact history. Individuals with uncomplicated scabies were provided with treatment in line with national guidelines using 25% benzyl benzoate emulsion. Those with bacterial superinfection were referred to the nearest health facility for additional antibiotic treatment. Health education on prevention of scabies transmission and timely treatment of the individuals was provided to the family.

### Data analysis

All the data were entered during the field work into a purpose-built Open Data Kit-based Android smartphone application. This application uses 256-bit encryption at rest and during transfer, providing industry-leading levels of security. The electronic survey form included GPS coordinates, the household and WASH questionnaires, individual demographic data, laboratory sample records, the scabies clinical examination tool, and GWD case search questions. The electronic data system included validation rules that prevented entry of out-of-range data, missed fields and other potential errors.

Data were analyzed using JAMOVI version 2.6 (https://www.jamovi.org) [30] and R version 4.5.1 (R Core Team, Vienna, Austria, https://www.r-project.org/) [31], both free open-source software. To account for the non-independence of individuals within the same household and households within the same cluster (per the study design), population prevalence percentages and their corresponding 95% confidence intervals (*CI*s) were computed using Taylor series linearization (robust sandwich estimators). To assess geographical variations in parasite distribution, differences in prevalence proportions across regions were evaluated for each species using the design-adjusted Rao-Scott second-order chi-square test of independence; species for which the total number of positives was <10 were excluded from this comparison. A mixed-effect regression model with cluster-level (5 regions) random effects was fitted to account for within-region variation of *T. solium* positivity. Given the survey design involved 20 clusters in each region (100 village clusters in total), a mixed effects regression model with the village clusters as the random effect was fitted to account for correlation between the clusters. Results are reported as adjusted odds ratios (*OR*s) with 95% *CI*.

The maps were generated using the free open-source QGIS version 3.44.0 (http://www.qgis.org) [32]. The map boundary data was obtained from the Humanitarian Data Exchange (HDX) managed by the United Nations Office for the Coordination of Humanitarian Affairs (OCHA).

## Results

### Identification of *T. solium* endemic or high-risk areas

**Fig. 2:**
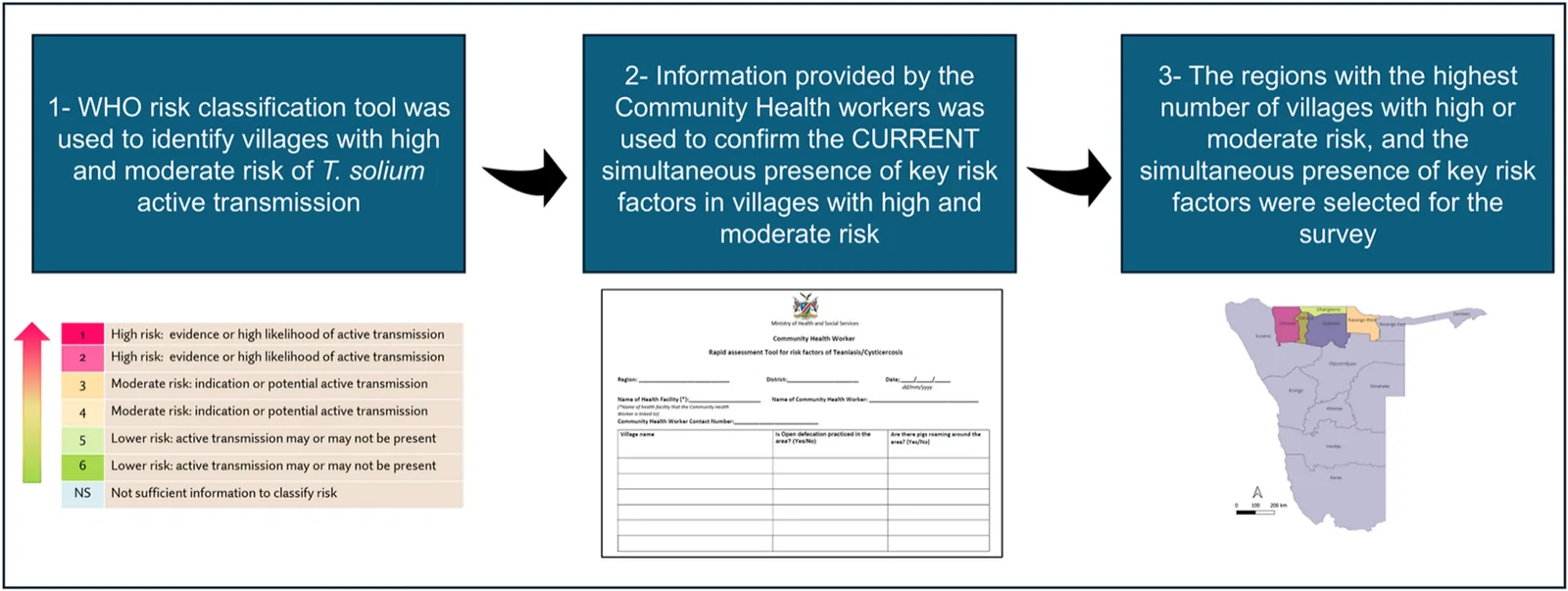
Steps used to identify and select the regions for the field survey

The 10 regions assessed in November 2023 with the WHO Risk Classification Tool were Zambezi, Kavango East, Kavango West, Ohangwena, Oshikoto, Oshana, Omusati, Kunene, Otjozondjupa and Omaheke. Based on all the information collected during the desk review, villages in those regions were classified as high, moderate or low risk of active *T. solium* transmission, and a total of 848 villages were identified as high or moderate risk (Table 1). They were also confirmed by the CHWs as having the current presence of both necessary risk factors for active transmission, that is roaming pigs and deficient sanitation. Five regions, Ohangwena, Oshana, Omusati, Oshikoto and Kavango West reported the highest number of villages with high or moderate risk, and the coexistence of current roaming pigs and deficient sanitation and were selected for the field surveys (Table 1, Figs. 1 and 2, and Supplementary file 3).

**Table 1:** Villages with reported risk factors for *Taenia solium* transmission at sub-district level

	Region	Number of villages with moderate risk (potential active transmission)	No. of villages with high risk (evidence or likelihood of active transmission)	Number of villages with moderate or high risk	Number of clusters visited per region	Number of households visited and interviewed
1	Ohangwena	247	0	247	20	520
2	Oshana	173	5	178	20	493
3	Omusati	170	0	170	20	469
4	Oshikoto	108	10	118	20	508
5	Kavango West	101	0	101	20	518
6	Kavango East	21	0	21	0	0
7	Zambezi	8	0	8	0	0
8	Kunene	2	0	2	0	0
9	Otjozondjupa	2	0	2	0	0
10	Omaheke	1	0	1	0	0
	Total	833	15	848	100	2508

### Survey results

The data collection phase was conducted from 1 July to 7th August 2024 in Kavango West, Ohangwena, Omusati, Oshana, and Oshikoto. A hundred clusters/villages were visited, 20 in each region. The households were identified through the community leadership of each cluster. A total of 2508 household questionnaires were administered to the heads of the households or their proxies in the five regions (Table 1). Most of the regions achieved the target number of 480 households per region except for Omusati region which reached 469 (97.7%).

In the households surveyed, most of the heads of the households interviewed were above 50 years old, and they had attended only up to primary school. Most of the heads of households and their proxy interviewed were female (Table 2).

**Table 2:** Demographic Information from the household survey

Region	Average age of household head	Gender^a^	Education			
			None	Primary	Secondary	Tertiary
Kavango West	51.5	M = 171 (33.0%) F = 347 (67.0%)	88 (17.0%)	253 (48.8%)	170 (32.8%)	7 (1.4%)
Ohangwena	56	M = 126 (24.2%) F = 394 (75.8%)	128 (24.6%)	209 (40.2%)	171 (32.9%)	12 (2.3%)
Omusati	56.5	M = 102 (21.7%) F = 367 (78.3%)	53 (11.3%)	303 (64.6%)	94 (20.0%)	19 (4.1%)
Oshana	57	M = 115 (23.3%) F = 378 (76.7%)	47 (9.5%)	238 (48.3%)	195 (39.6%)	13 (2.6%)
Oshikoto	55	M = 107 (21.1%) F = 401 (78.9%)	46 (9.1%)	223 (43.9%)	203 (40.0%)	36 (7.1%)
All regions		M = 621 (24.8%) F = 1887 (75.2%)	362 (14.4%)	1226 (48.9%)	833 (33.2%)	87 (3.5%)

^a^*M* male, *F* female

A total of 2307 responses were obtained regarding the main water sources used in households. The results showed variations within regions (Table 3). The largest % of households in Omusati, Oshana, and Oshikoto had piped water into their dwelling, while in Ohangwena the largest proportion had piped water to the yard or compound. In Kavango West, the largest percentage of households obtained water from public taps, tubewells or boreholes.

**Table 3:** Main safe water sources and sanitation structures available to households per region

Water sources	Total households	Kavango West	Ohangwena	Omusati	Oshana	Oshikoto
Piped water into dwelling	938 (40.7%)	47 (11.6%)	–	207 (47.0%)	391 (80.0%)	293 (58.7%)
Piped water to neighbor	346 (15.0%)	16 (4.0%)	60 (12.6%)	180 (40.9%)	46 (9.4%)	44 (8.8%)
Piped water to yard, compound, or plot	408 (17.7%)	32 (7.9%)	213 (44.8%)	–	40 (8.2%)	123 (24.6%)
Public tap/standpipe	361 (15.6%)	156 (38.6%)	147 (30.9%)	21 (4.8%)	11 (2.2%)	26 (5.2%)
Tubewell/borehole	198 (8.6%)	152 (37.6%)	14 (2.9%)	29 (6.6%)	–	3 (0.6%)
Protected dug well	56 (2.4%)	1 (0.2%)	41 (8.6%)	3 (0.7%)	1 (0.2%)	10 (2.0%)
Sanitation structures	Total households	Kavango West	Ohangwena	Omusati	Oshana	Oshikoto
Shared or public latrine	17 (0.7%)	8 (1.5%)	1 (0.2%)	2 (0.4%)	6 (1.2%)	0
Private latrine	904 (36.0%)	13 (2.5%)	120 (23.1%)	195 (41.6%)	300 (60.9%)	276 (54.3%)
No structure, defecation outside	1529 (61.0%)	496 (95.8%)	368 (70.8%)	268 (57.1%)	165 (33.5%)	232 (45.7%)
Other	58 (2.3%)	1 (0.2%)	31 (6.0%)	4 (0.9%)	22 (4.5%)	0

Sanitation structures	Total households	Kavango West	Ohangwena	Omusati	Oshana	Oshikoto
Shared or public latrine	17 (0.7%)	8 (1.5%)	1 (0.2%)	2 (0.4%)	6 (1.2%)	0
Private latrine	904 (36.0%)	13 (2.5%)	120 (23.1%)	195 (41.6%)	300 (60.9%)	276 (54.3%)
No structure, defecation outside	1529 (61.0%)	496 (95.8%)	368 (70.8%)	268 (57.1%)	165 (33.5%)	232 (45.7%)
Other	58 (2.3%)	1 (0.2%)	31 (6.0%)	4 (0.9%)	22 (4.5%)	0

Number of households (and frequencies)

Regarding sanitation structures, from over 2508 responding households, a total of 61% reported no sanitation structure and only 36% of the households had a private latrine (Table 3). There was variation between regions. Kavango West region reported the lowest presence of sanitation structures followed by Ohangwena and Omusati region.

From a total of 2508 households, 41% reported keeping pigs in the household (Table 4). There was a variation in the percentage of households allowing the pigs to roam between regions. In all regions most households ate pork, from pigs that were bred at home or obtained from the local slaughterhouses. Most of the households reported consuming boiled or cooked pork (Table 4).

**Table 4:** Household information on pigs

Questions	Answers	Kavango West	Ohangwena	Omusati	Oshana	Oshikoto		Total
Do you have pigs in the yard/homestead?	No	464 (89.6%)	317 (61.0%)	156 (33.3%)	247 (50.1%)	295 (58.1%)	1479 (59.0%)	
	Yes	54 (10.4%)	203 (39.0%)	313 (66.7%)	246 (49.9%)	213 (41.9%)	1029 (41.0%)	
If yes, how do they get food?	They are fed while in the pen/yard	37 (68.5%)	81 (39.9%)	242 (77.3%)	179 (72.8%)	198 (93.0%)	737 (71.6%)	
	They roam around to look for food	2 (3.7%)	82 (40.4%)	60 (19.2%)	48 (19.5%)	15 (7.0%)	207 (20.1%)	
	Both	15 (27.8%)	40 (19.7%)	11 (3.5%)	19 (7.7%)	0	85 (8.3%)	
Do you eat pork?	No	109 (21.0%)	29 (5.6%)	4 (0.9%)	23 (4.7%)	23 (4.5%)	188 (7.5%)	
	Yes	409 (79.0%)	491 (94.4%)	465 (99.1%)	470 (95.3%)	485 (95.5%)	2320 (92.5%)	
If yes, what is the main source of your pork?	Home	3 (0.7%)	194 (39.5%)	140 (30.1%)	12 (2.6%)	78 (16.1%)	427 (18.4%)	
	Local slaughter houses	403 (98.5%)	246 (50.1%)	301 (64.7%)	17 (3.6%)	378 (77.9%)	1345 (58.0%)	
	Butcher shop	3 (0.7%)	51 (10.4%)	12 (2.6%)	220 (46.8%)	29 (6.0%)	315 (13.6%)	
	Other	0	0	12 (2.6%)	221 (47.0%)	0	233 (10.0%)	
How do you prepare your pork for consumption?	Eat raw	0	0	0	0	0	0	
	Grill	1 (0.2%)	5 (1.0%)	1 (0.2%)	2 (0.4%)	5 (1.0%)	14 (0.6%)	
	Boil/cook	408 (99.8%)	485 (98.8%)	464 (99.8%)	466 (99.1%)	480 (99.0%)	2303 (99.3%)	
	Other	0	1 (0.2%)	0	2 (0.4%)	0	3 (0.1%)	

Number of households and frequencies

### Taeniasis, schistosomiasis and other helminths results

A total of 4880 stool samples and 4876 urine samples were collected. More females than males, and more adults than children provided stool and urine samples (Table 5).

**Table 5:** Age breakdown of people providing stool and urine samples

Region	Sample type	Total	Male 5–17 years	Female 5–17 years	Male >18 years	Female >18 years
Kavango West	Stool	972	139 (14.3%)	200 (20.6%)	196 (20.2%)	437 (45.0%)
	Urine	970	137 (14.1%)	200 (20.6%)	196 (20.2%)	437 (45.1%)
Ohangwena	Stool	992	190 (19.2%)	205 (20.7%)	132 (13.3%)	465 (46.9%)
	Urine	990	190 (19.2%)	204 (20.6%)	132 (13.3%)	464 (46.9%)
Omusati	Stool	932	186 (20.0%)	204 (21.9%)	113 (12.1%)	429 (46.0%)
	Urine	932	186 (20.0%)	204 (21.9%)	113 (12.1%)	429 (46.0%)
Oshana	Stool	981	155 (15.8%)	187 (19.1%)	144 (14.7%)	495 (50.5%)
	Urine	981	155 (15.8%)	187 (19.1%)	144 (14.7%)	495 (50.5%)
Oshikoto	Stool	1003	184 (18.3%)	244 (24.3%)	104 (10.4%)	471 (47.0%)
	Urine	1003	184 (18.3%)	244 (24.3%)	104 (10.4%)	471 (47.0%)
Total	Stool	4880	854 (17.5%)	1040 (21.3%)	689 (14.1%)	2297 (47.1%)
	Urine	4876	852 (17.5%)	1039 (21.3%)	689 (14.1%)	2296 (47.1%)
Total children and adults	Stool	4880	1894 (38.8%)		2986 (61.2%)	
	Urine	4876	1891 (38.8%)		2985 (61.2%)	

Numbers and percentages across the regions

The samples were processed in the NIP laboratory using the Kato–Katz and urine filtration for fecal and urine samples respectively. A total of 20 samples were positive for taeniasis, which were sent to the University of Melbourne for species confirmation. Molecular analyses confirmed the *Taenia* species involved in 17 of the egg-positive samples. Twelve cases were confirmed as *T. solium* and five as *T. saginata* (Tables 6 and 7). The three Kato–Katz positive samples negative to PCR, were one from Oshana, and two from Kavango West. *T. solium* positive samples were found in all regions except for Oshana. There was no statistically significant difference in the positivity rate for *T. solium* between regions (Rao–Scott second-order chi-square test of independence test *P* = 0.112). The only region in which the positivity rate exceeded the WHO 0.5% threshold was Ohangwena with 0.60%, but in Oshikoto it is included in the 95% *CI *(0.00–0.62%).

**Table 6:** Number of positive samples for taeniasis, schistosomiasis and soil-transmitted helminths per region (percentage, 95%* CI*)

	Kavango West	Ohangwena	Omusati	Oshana	Oshikoto	Total
Number tested	972	992	932	981	1003	4880
*Taenia solium*						
Positive^a^	2	6	1	0	3	12
Prevalence	0.21%	0.60%	0.11%	0.00%	0.30%	
(95% CI)	(0.00–0.48%)	(0.00–1.22%)	(0.00–0.31%)	(0.00–0.00%)	(0.00–0.62%)	
*T. saginata*						
Positive	0	0	1	3	1	5
Prevalence	0.00%	0.00%	0.11%	0.31%	0.10%	
(95% *CI*)	(0.00–0.00%)	(0.00–0.00%)	(0.00–0.31%)	(0.00–0.73%)	(0.00–0.30%)	
*Schistosoma haematobium*						
Positive^b^	97	3	3	1	7	111
Prevalence	9.98%	0.30%	0.32%	0.10%	0.70%	
(95% *CI*)	(2.69–17.07%)	(0.00–0.62%)	(0.00–0.67%)	(0.00–0.30%)	(0.07–1.32%)	
*S. mansoni*						
Positive^c^	16	0	0	0	0	16
Prevalence	1.65%	0.00%	0.00%	0.00%	0.00%	
(95% *CI*)	(0.26–3.03%)	(0.00–0.00%)	(0.00–0.00%)	(0.00–0.00%)	(0.00–0.00%)	
*S. intercalatum*^d^						
Positive	0	0	0	0	1	1
Prevalence	0.00%	0.00%	0.00%	0.00%	0.10%	
(95% CI)	(0.00–0.00%)	(0.00–0.00%)	(0.00–0.00%)	(0.00–0.00%)	(0.00–0.29%)	
Hookworms						
Positive^e^	46	4	2	2	78	132
Prevalence	4.73%	0.40%	0.21%	0.20%	7.78%	
(95% CI)	(2.85–6.61%)	(0.00–0.89%)	(0.00–0.50%)	(0.00–0.48%)	(5.02–10.53%)	
*Ascaris lumbricoides*						
Positive	9	0	0	0	0	9
Prevalence	0.93%	0.00%	0.00%	0.00%	0.00%	
(95% CI)	(0.32–1.53%)	(0.00–0.00%)	(0.00–0.00%)	(0.00–0.00%)	(0.00–0.00%)	
*Trichuris trichiura*						
Positive	0	0	0	1	0	1
Prevalence	0.00%	0.00%	0.00%	0.10%	0.00%	
(95% *CI*)	(0.00–0.00%)	(0.00–0.00%)	(0.00–0.00%)	(0.00–0.30%)	(0.00–0.00%)	
*Enterobius vermicularis*						
Positive^f^	0	3	0	1	8	12
Prevalence	0.00%	0.30%	0.00%	0.10%	0.80%	
(95% *CI*)	(0.00–0.00%)	(0.00–0.63%)	(0.00–0.00%)	(0.00–0.30%)	(0.17–1.43%)	
*Fasciola* spp.						
Positive	4	0	0	0	0	4
Prevalence	0.41%	0.00%	0.00%	0.00%	0.00%	
(95% *CI*)	(0.00–0.88%)	(0.00–0.00%)	(0.00–0.00%)	(0.00–0.00%)	(0.00–0.00%)	
*Hymenolepis nana*						
Positive^g^	25	6	17	8	9	65
Prevalence	2.57%	0.60%	1.82%	0.82%	0.90%	
(95%* CI*)	(1.61–3.54%)	(0.04–1.17%)	(0.88–2.76%)	(0.40–1.23%)	(0.34–1.46%)	
*Diphyllobothrium latum*						
Positive	1	0	0	0	0	1
Prevalence	0.10%	0.00%	0.00%	0.00%	0.00%	
(95% *CI*)	(0.00–0.30%)	(0.00–0.00%)	(0.00–0.00%)	(0.00–0.00%)	(0.00–0.00%)	

*P* value for the difference between regions is based on Rao–Scott second-order chi-square test of independence species; species with < 10 positive cases were excluded

^a^*P* = 0.112, ^b, c, e, g^*P* < 0.001, ^f^*P* = 0.04

^d^It was presumed to be *S. intercalatum* but no additional tests were conducted to differentiate from *S. bovis* or hybrid species

**Table 7:** Samples identified as Kato–Katz *Taenia* egg positive and tapeworm species confirmed by molecular tests

Sample ID	Sex	Age	Kato–Katz *T. solium* egg count	PCR/sequence result
1	Female	72	9	*T. solium*
2	Female	48	199	*T. solium*
3	Female	27	61	*T. solium*
4	Female	35	29	*T. solium*
5	Female	10	13	*T. solium*
6	Female	36	109	*T. solium*
7	Male	49	30	*T. saginata*
8	Female	28	38	*T. solium*
9	Male	60	4	*T. saginata*
10	Female	30	1	Negative
11	Male	60	6	*T. saginata*
12	Male	12	15	*T. saginata*
13	Male	12	11	*T. solium*
14	Female	42	234	*T. saginata*
15	Female	21	16	*T. solium*
16	Male	17	36	*T. solium*
17	Female	47	45	*T. solium*
18	Female	28	1	Negative
19	Female	6	3	Negative
20	Male	20	100	*T. solium*

The mixed-effects logistic regression with region as a random effect (5 clusters) yielded a region variance of 18.98, suggesting evidence of clustering at the regional level. The model showed no significant association between *T. solium* positivity and age-group (*OR* = 0.52, 95%* CI:* 0.15–1.84, *P* = 0.313) or sex (*OR* = 0.55, 95% *CI:* 0.14–2.15, *P* = 0.392). A second model with village clusters as a random effect (82 of the 100 clusters; 18 clusters had no stool samples and were excluded) showed village-cluster variance of 73.49, confirming clustering at the village level. After accounting for this clustering, neither age group (*OR* = 0.403, 95% *CI*: 0.0971–1.67, *P* = 0.210) nor sex (*OR* = 0.57, 95%* CI*: 0.124–2.63, *P* = 0.472) were significantly associated with *T. solium* positivity.

The median egg count for *T. solium* (median = 37, IQR = 55.5) was not significantly different (Mann–Whitney U test) from the median egg count for *T. saginata* (median = 15, IQR = 24.0) (U = 20.0, *P* = 0.328), and that the median age for the patients with *T. solium* (median = 27.5, IQR = 19.5) was not significantly different from the median age for the patients with *T. saginata* (median = 49, IQR = 18.0) (U = 16.5, *P* = 0.170).

**Fig. 3:**
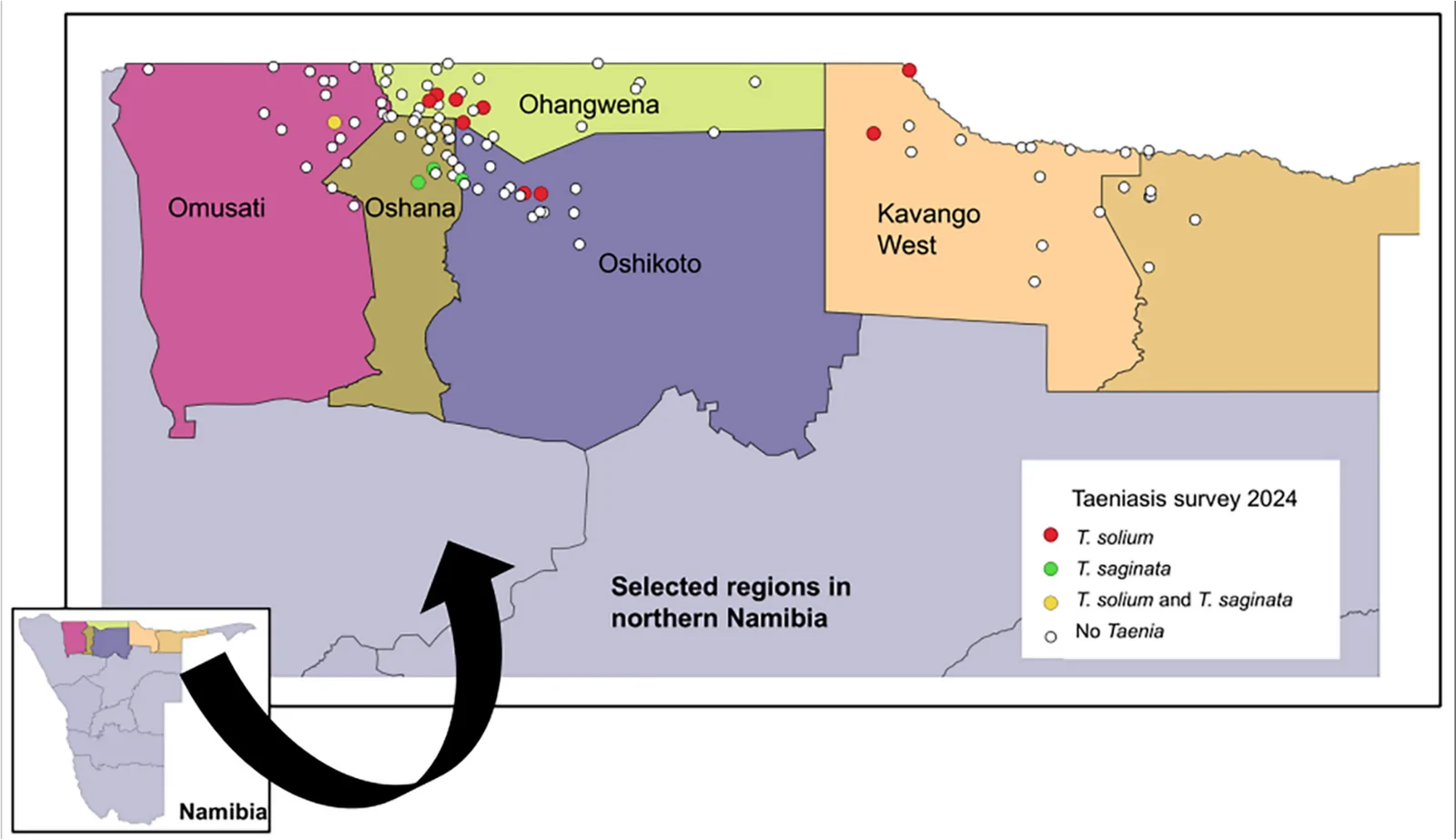
Villages tested and those with positive results for *T. solium* and *T. saginata*. Map generated using QGIS 3.44.0 (QGIS Development team in https://www.qgis.org/). The map boundary data was obtained from the Humanitarian Data Exchange (HDX) managed by the United Nations Office for the Coordination of Humanitarian Affairs (OCHA) and made available under a Creative Commons Attribution 4.0 (https://data.humdata.org/dataset/cod-ab-nam)

*T. solium* was reported from four villages in Ohangwena region, i.e., Onamahoka 5.7% (2/35), Okafitu 5.6% (2/36), Oshali 2.6% (1/38) and Epalala 1.8% (1/56). It was also reported from three villages in Oshikoto region, i.e., Omangundu 1.7% (1/59), Oniika 2.8% (1/36) and Okadhiya 1.7% (1/60); 2 villages from Kavango West, i.e., Nkoro 1.8% (1/57) and Kwaki 1.8% (1/55); and one village from Omusati, Okadhiyakohumba 1.7% (1/60). *T. saginata* was found in two villages in Oshana region, i.e., Onkama 3.3% (2/61) and Olwege 1.7% (1/60), one village in Oshikoto, Uukelo 4.2% (1/24), and one village in Omusati region, Okadhiyakohumba 1.7% (1/60). The distribution of the villages tested, the villages positive for *T. solium* and *T. saginata* is shown in Fig. 3.

A total of 128 samples were positive for schistosomiasis. One hundred and eleven (111) cases were *S. haematobium*, 16 cases *S. mansoni*, and one for *S. intercalatum* (Table 6). *S. haematobium* was found in all regions, with a high positivity rate in Kavango West (9.98%), and statistically significant differences between regions (Rao–Scott second-order chi-square test of independence test *P* < 0.001). *S. mansoni* was only found in Kavango West (1.65%) and it differed significantly between regions (Rao–Scott second-order chi-square test of independence test *P* < 0.001). The total prevalence of schistosomiasis in Kavango West was 11.63% (113 positives out of 972). One sample was presumed to be *S. intercalatum*, but no additional tests were conducted to differentiate it from *S. bovis* or hybrid species.

**Fig. 4:**
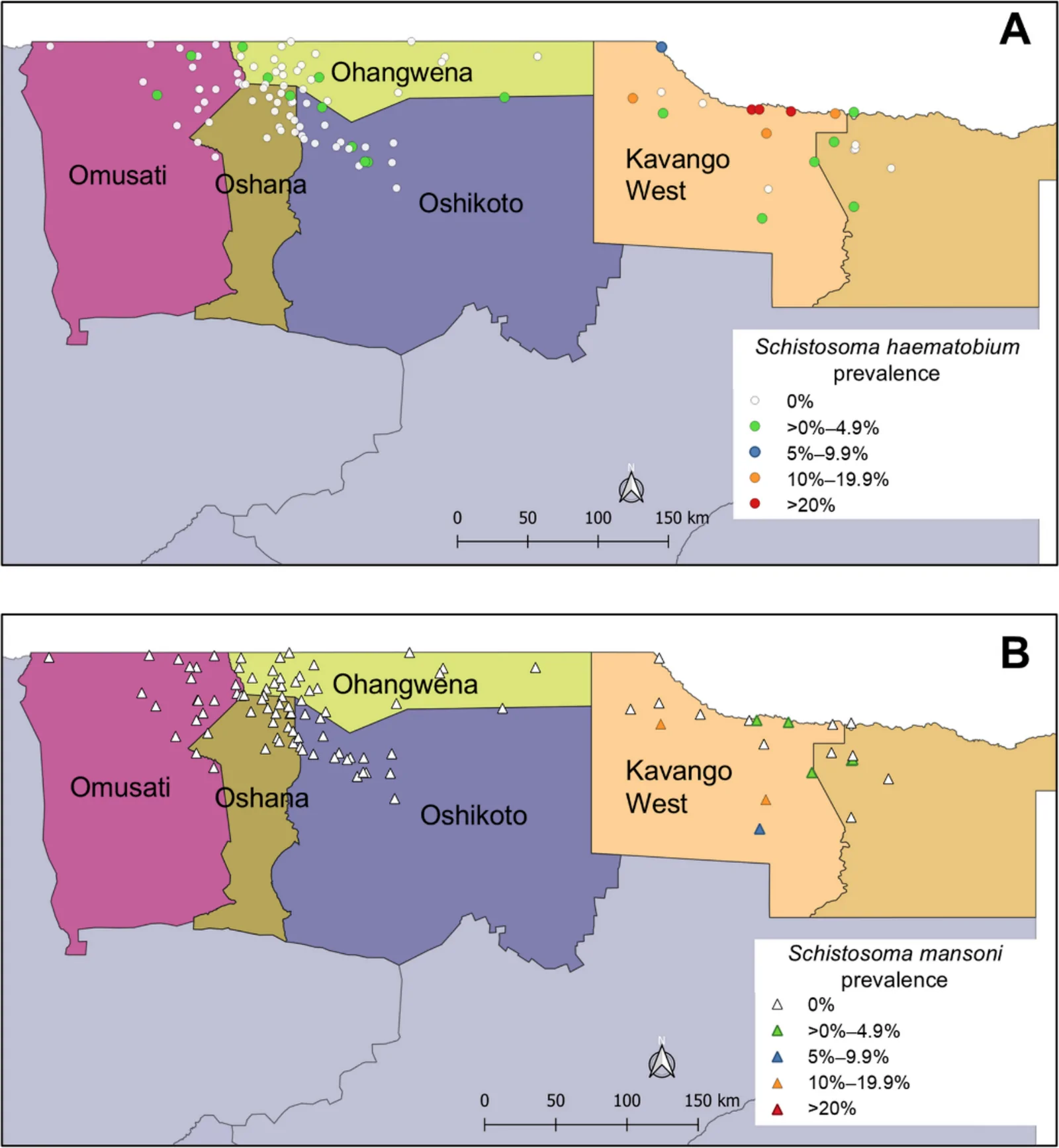
Villages tested and prevalence results for *S. haematobium* (**A**) and *S. mansoni* (**B**). Map generated using QGIS 3.44.0 (QGIS Development team in https://www.qgis.org/). The map boundary data was obtained from the Humanitarian Data Exchange (HDX) managed by the United Nations Office for the Coordination of Humanitarian Affairs (OCHA) and made available under a Creative Commons Attribution 4.0 (https://data.humdata.org/dataset/cod-ab-nam)

The distribution of the villages tested, the villages positive for *S. mansoni* and *S. haematobium,* are shown in Fig. 4.

Schistosomiasis prevalences were compared with the ones obtained by Sousa-Figueiredo et al. [3] in 2012–2013 (Table 8).

**Table 8:** Comparison of schistosomiasis prevalence between the surveys conducted in 2012–2013 [3] and the 2024 survey

Region	Number samples tested		*Schistosoma haematobium*			*S. mansoni*		
	2012–2013	2024	2012–2013	2024	2024 (SAC only)	2012–2013	2024	2024 (SAC only)
Kavango^a^	4538	972	8.1%	10.0%	17.4%	11.5%	1.6%	0.7%
Ohangwena	3479	992	3.0%	0.3%	0	1.2%	0	0
Omusati	3880	932	4.1%	0.3%	0.3%	1.0%	0	0
Oshana	1919	981	5.6%	0.1%	0	1.3%	0	0
Oshikoto	2700	1003	2.9%	0.7%	0.8%	0.7%	0	0

*SAC* school aged children. The 2012–2013 survey was conducted in SAC only, while in 2024 included children and adults. The 2024 data are presented for the total population and SAC only

^a^In 2012–2013, Kavango included Kavango West and Kavango East. In 2024, it was mainly Kavango West

Other helminths of interest identified were hookworms, *A. lumbricoides, T. trichiura, E. vermicularis, Fasciola* spp.*, H. nana* (also known as *Rodentolepis nana*) and *D. latum* (Table 6).

### Scabies assessment results

A total of 7707 household participants were clinically assessed for scabies. There were 150 (1.9%) participants who were classified positive for scabies using the IACS criteria. Statistically significant differences in prevalence were observed between regions (Table 9). The majority of cases, 88 (6.6%), were recorded in Ohangwena region.

**Table 9:** Prevalence of scabies per region

	Kavango West	Ohangwena	Omusati	Oshana	Oshikoto	Total
Total assessed	1609	1339	1818	1209	1732	7707
Negative for scabies	1602	1251	1804	1186	1714	7557
Positive for scabies	7	88	14	23	18	150
Prevalence of clinical Scabies	0.4%	6.6%	0.8%	1.9%	1.0%	1.9%
Chi-squared test of independence						*X*^2^ = 190, *P* < 0.001

**Fig. 5:**
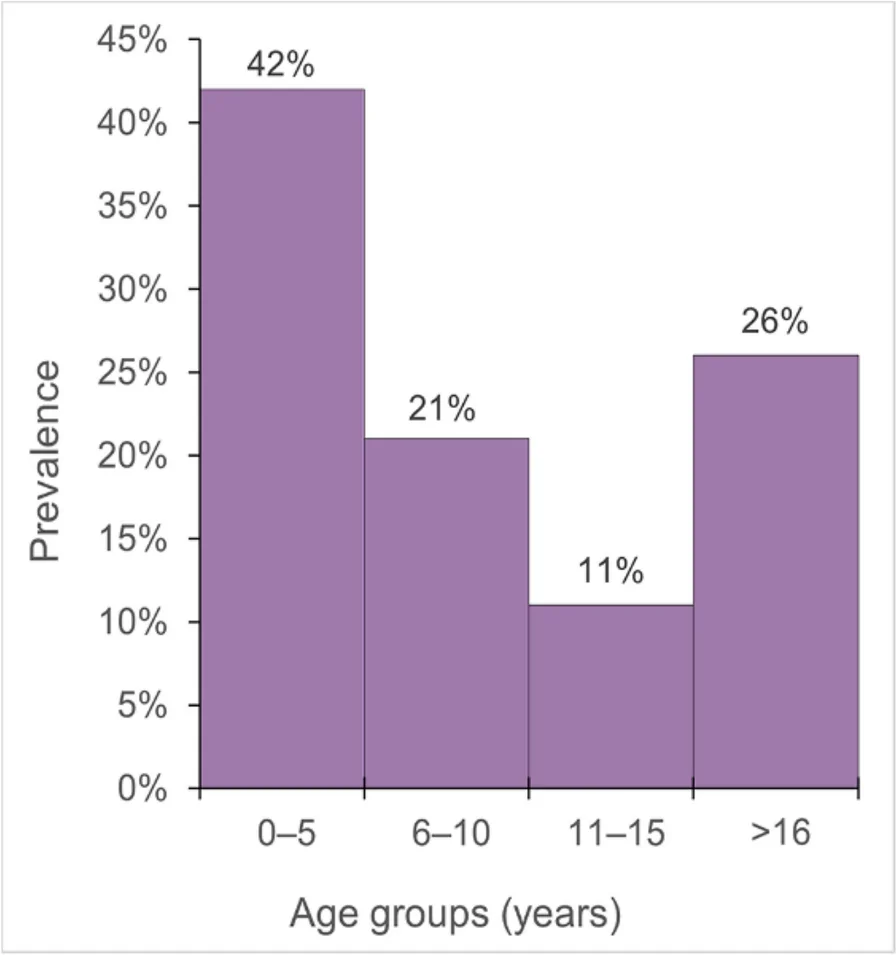
Scabies prevalence in the different age groups

The highest percentage of scabies cases was recorded among children between 0 and 5 years of age, followed by the group of 16 years and above, the 6–10 years old group and lastly the 11–15 years of age (Fig. 5).

All the positive scabies cases and their contacts were given treatment at the time of the survey with Benzyl Benzoate according to the Namibia standard treatment guidelines, second edition [33].

### Guinea-worm disease results

The majority of respondents 99.8% (*n* = 2505) in all regions indicated that they had not seen the Guinea worm in a human. However, three respondents (0.2%) indicated that they had seen the worm, all from Ohangwena Region (Table 10). One respondent also from Ohangwena reported having seen the worm in a dog.

**Table 10:** Household information on Guinea-worm disease

Questions		Kavango West	Ohangwena	Omusati	Oshana	Oshikoto	Total
(1) Did you see the worm shown in the picture in a human (ONLY) in the past 12 months?	Yes	0	3 (0.58%)	0	0	0	3 (0.12%)
	No	518 (100%)	517 (99.4%)	469 (100%)	493 (100%)	508 (100%)	2505 (99.88%)
(2) If YES to 1 above, specify:	Area or district		Okahala, Okambada, Oshixunga				Okahala, Okambada, Oshixunga
	Village		Angola, Okambada, Oshixunga				Angola, Okambada, Oshixunga
(3) Did you see the worm shown in the picture in a dog or other animal (ONLY) in the past 12 months?	Yes	0	1 (0.19%)	0	0	0	1 (0.04%)
(4) If YES to 3 above, specify:	Which animal		Dog				Dog
	Which area		Oshixunga				Oshixunga

## Discussion

Five regions were identified as high risk for *T. solium* using the WHO Risk Classification Tool, and field surveys were conducted in each including Ohangwena, Oshana, Omusati, Oshikoto and Kavango West, to confirm endemicity for *T. solium*. The key risk factors for *T. solium* transmission are the simultaneous presence of roaming pigs and deficient sanitation, and all regions confirmed the presence of both risk factors. The coexistence of roaming pigs and inadequate sanitation observed is consistent with findings from other endemic settings in sub-Saharan Africa. Similar associations have been reported in Tanzania [34], Zambia [35] and other African countries [36].

Three Kato–Katz positive samples for taeniasis tested negative on PCR undertaken to identify the *Taenia* species involved. Those three samples had the lower number of eggs (two samples with one egg, and one sample with three eggs), suggesting an insufficient quantity of *Taenia*-derived DNA or possibly that they were false positives in Kato–Katz testing.

The survey results need to be interpreted for each region as that was the intention of the survey design. As such, only Ohangwena surpassed the 0.5% threshold suggested by the WHO to trigger a public health intervention, and ideally, a One Health intervention should be implemented addressing the transmission in humans and pigs to have a faster impact [37]. Although only Ohangwena exceeded the WHO threshold for triggering a public health response, the identification of *T. solium* in neighboring regions suggests focal but potentially connected transmission zones. Studies in Latin America and sub-Saharan Africa [38, 39] have demonstrated marked spatial clustering of *T. solium* infections at household and village level, and the close geographic proximity of positive villages across administrative boundaries in northern Namibia suggests that transmission may similarly occur in clustered microfoci rather than being uniformly distributed across regions.

The findings in Ohangwena correlate with the identification of serious adverse reaction during the MDA campaign of 2019. However, *T. solium* was also identified in Oshikoto (an area in which the 95% *CI* includes the threshold), Omusati and Kavango West, which means that precautions need to be taken when using MDA with praziquantel to avoid neurological adverse events in those regions, especially in the communities in which pigs are kept. Oshana does not have any positive identification for *T. solium*, however many villages are very close to the cluster of positives in Ohangwena and one of the positive villages in Oshikoto. As there are no natural barriers between those communities, and the movement of pigs and people is possible in the area, it will also be warranted to implement precautions when using praziquantel for MDA in the neighboring areas. Precautions taken in recent *T. solium* control programs aim to identify people with symptoms and signs compatible with NCC and treat people at risk with niclosamide instead, as well as early identification and adequate treatment of neurological adverse events [15]. These findings highlight the importance of understanding *T. solium* endemicity and strengthening pharmacovigilance and preparedness during praziquantel MDA campaigns in co-endemic areas.

The presence of *S*. *haematobium* was confirmed in all regions. *S. mansoni* was only identified in Kavango West with a low prevalence. Comparing the prevalences to the ones obtained by Sousa-Figueiredo et al. [3] in 2012–2013, current prevalences are lower, except for *S. haematobium* in Kavango (Table 8). Important differences between the surveys include that in 2012–2013, the survey was conducted in school-aged children, while in 2024 in all the population, and in the first survey a rapid diagnostic test for the schistosome cathodic antigen (CCA) was used for *S. mansoni*, and Hemastix reagent strip was used for *S. haematobium* alongside the Kato–Katz and the urine filtration tests for assessment of diagnostic performance. Previous surveys have documented schistosomiasis endemicity in northern Namibia, including in areas overlapping with the regions assessed in the present study. Overall reductions in schistosomiasis prevalence following repeated praziquantel MDA have been reported from several countries in the African region [40, 41] including Mozambique, Tanzania [42] and Uganda [43]. However, studies from these settings have also shown persistent, localized hotspots despite overall reductions in prevalence, particularly in communities with continued exposure to infested water bodies and limited access to water and sanitation services. The persistence of higher prevalence in Kavango West may therefore reflect ongoing focal transmission requiring targeted surveillance and sustained intervention efforts.

The overall prevalence of schistosomiasis in Kavango West was 11.63% (i.e., 9.98% for *S. haematobium* + 1.65% for *S. mansoni* in 2024) which is above the threshold recommended by WHO for implementing MDA [44].

The prevalence of other helminths was low (Table 6); however, the results should be interpreted with care. Delays between sample collection and Kato–Katz testing might underestimate hookworm prevalence [26, 45], and the surveyed population included children and adults, when traditional surveys only include children as they are the most vulnerable and have the highest risk of acquiring soil-transmitted infections [46].

The scabies prevalence in all regions was below the threshold of 10% required for MDA to be highly effective [47]. Besides the treatment conducted at the time of the survey, these findings indicate that intensified control measures may no longer be necessary; but ongoing public health interventions, including health education, community engagement, surveillance, and case management, should be maintained to sustain the gains achieved. However, according to the DHIS2 2020 to 2023 reports on scabies, Ohangwena region tops the list of scabies cases compared to other regions. The factors contributing to this situation require further investigation.

Our analysis also showed that scabies cases were higher (42%) in children between 0 and 5 years compared to other age groups. This is similar to findings in studies conducted in Ethiopia, Nigeria and at global level [48–50] where transmission is often concentrated among preschool- and school-aged children due to close physical contact and overcrowded living conditions.

Except for 3 households in Ohangwena, there were no reports of having seen GWD in humans or dogs. Ohangwena region is bordering Cunene province of Angola where cases of GWD have been reported between 2018 and 2020 [20]. Consequently, further investigation is needed in the area.

There were some limitations that affected the performance of the work described: limited information was available for the risk analysis from the animal side, the random selection of households within a village was changed to a snowball sampling technique due to the challenges in the field, and the shortage of staff in NIP laboratories during the first 3 weeks slowed down the processing of laboratory analysis of samples. Additionally, due to limited financial resources, the preferred practice of preparing two slides per sample for Kato–Katz for laboratory analysis could not be implemented.

Further to the technical results obtained for each of the diseases targeted in this integrated survey, some important lessons were learned. The key lessons include the importance of having enough financial and human resources to conduct the surveys as planned, allowing flexibility in any funded survey to integrate several other critical public surveys, the need to strengthen community mobilization during sensitization sessions to avoid refusals, and consideration to the survey timings as the household survey was conducted during school term and most children could not be reached as they attended classes.

The original surveys were aimed at determining the endemicity of *T. solium*. This was the first time that such surveys were conducted in Namibia. Calls have been made for integration of NTDs in the spectrum of program activities including planning, mapping, training, logistics, education and social mobilization, surveillance, etc. [51]. Considering the remoteness of the area and the limited resources and opportunities, it seemed sensible to take the opportunity to integrate surveys for other neglected diseases. However, the integration added complexity, additional resources and time were required compared to dealing with only one disease, and efficient coordination was essential. It is important that the funders (donors or governments) understand the benefits of integration but also understand and account for the added complexity and resources needed.

The integrated survey was also especially important for Namibia’s schistosomiasis control program, as by identifying infection with *T. solium* alongside schistosomiasis, the survey helps programs anticipate and manage MDA-associated risks in co-endemic settings, reinforcing the value of integrated NTD surveillance. In the case of Namibia, this data provides a basis for resuming the schistosomiasis control program, now guided by a targeted strategy to prevent and manage potential adverse events associated with praziquantel treatment in co-infected individuals [15], based on the co-endemicity determined in this survey.

Few published studies have documented integrated surveys simultaneously assessing *T. solium*, schistosomiasis, scabies and Guinea-worm surveillance within a single operational platform [52]. The Namibia experience therefore demonstrates the feasibility and added programmatic value of integrated community-based surveillance in remote endemic settings.

## Conclusions

Evidence was obtained concerning the presence of *T. solium* transmission in northern Namibia which enables the MoHSS to develop integrated control programs for *T. solium* and schistosomiasis. New information was obtained on scabies and GWD in the survey regions. The success of Namibia's integrated approach offers a model for other countries at early stages of implementing *T. solium* control programs, demonstrating how strategic collaboration, program integration and comprehensive assessment can optimize resource utilization and assist on the development and implementation of public health programs.

## Supplementary Information

Additional file 1

Additional file 2

Additional file 3

## Data Availability

The datasets used and analysed during the current study are available from the corresponding authors on reasonable request.

## Abbreviations

*CI*: Confidence interval
GWD: Guinea-worm disease
HDX: Humanitarian Data Exchange
MDA: Mass drug administration
MoHSS: Ministry of Health and Social Services of Namibia
NCC: Neurocysticercosis
NIP: Namibian Institute of Pathology
NTDs: Neglected Tropical Diseases
OCHA: United Nations Office for the Coordination of Humanitarian Affairs
*OR*: Odds ratio
PCR: Polymerase chain reaction
WASH: Water, sanitation and hygiene
WHO: World Health Organization
