# Supplementary material for: Integrated survey of Taenia solium endemicity, schistosomiasis, scabies, and Guinea-worm disease in northern Namibia
Source: Infect Dis Poverty. 2026 Nov 3;15:111. doi: 10.1186/s40249-026-01514-w (PMC13729652; doi:10.1186/s40249-026-01514-w)
Supplement: Supplementary file 1 [file 40249_2026_1514_MOESM1_ESM.pdf]

Ministry of Health and Social Services

Community Health Worker

Rapid assessment Tool for risk factors of Teaniasis/Cysticercosis

Region: ______________________________ **District:**________________________ **Date:**____/_____/_____
dd/mm/yyyy

Name of Health Facility (*):________________________ **Name of Community Health Worker:** ____________________________________
*(*Name of health facility that the Community Health Worker is linked to)*

Community Health Worker Contact Number:____________________________

Village name	Is Open defecation practiced in the area? (Yes/No)	Are there pigs roaming around the area? (Yes/No)

Ministry of Health and Social Services

Health Facility Summary

Rapid assessment Tool for risk factors of Teaniasis/Cysticercosis

Region: ______________________________ **District:**____________________________________ **Date:**_____/_____/_____
dd/mm/yyyy

Name of Health Facility:_______________________________ **Name of reporting health worker:**_______________________________________

Contact Number:____________________________________

Name of Community Health Worker	Community Health Worker Contact number	Village name	Is Open defecation practiced in the area? (Yes/No)	Are there pigs roaming around the area? (Yes/No)
