# Supplementary material for: Integrated survey of Taenia solium endemicity, schistosomiasis, scabies, and Guinea-worm disease in northern Namibia
Source: Infect Dis Poverty. 2026 Nov 3;15:111. doi: 10.1186/s40249-026-01514-w (PMC13729652; doi:10.1186/s40249-026-01514-w)
Supplement: Supplementary file 2 [file 40249_2026_1514_MOESM2_ESM.pdf]

DRACUNCULIASIS

World Health Organization

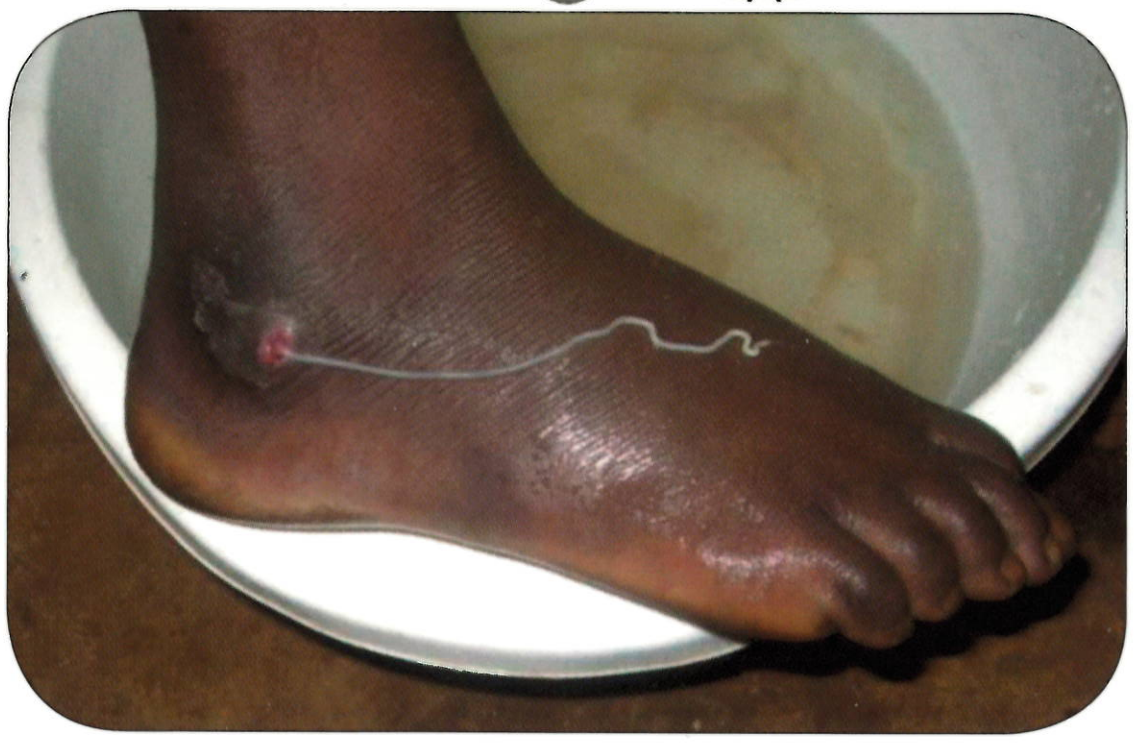

STOP GUINEA WORM DISEASE NOW
IF YOU SEE THIS, INFORM YOUR HEALTH WORKER IMMEDIATELY

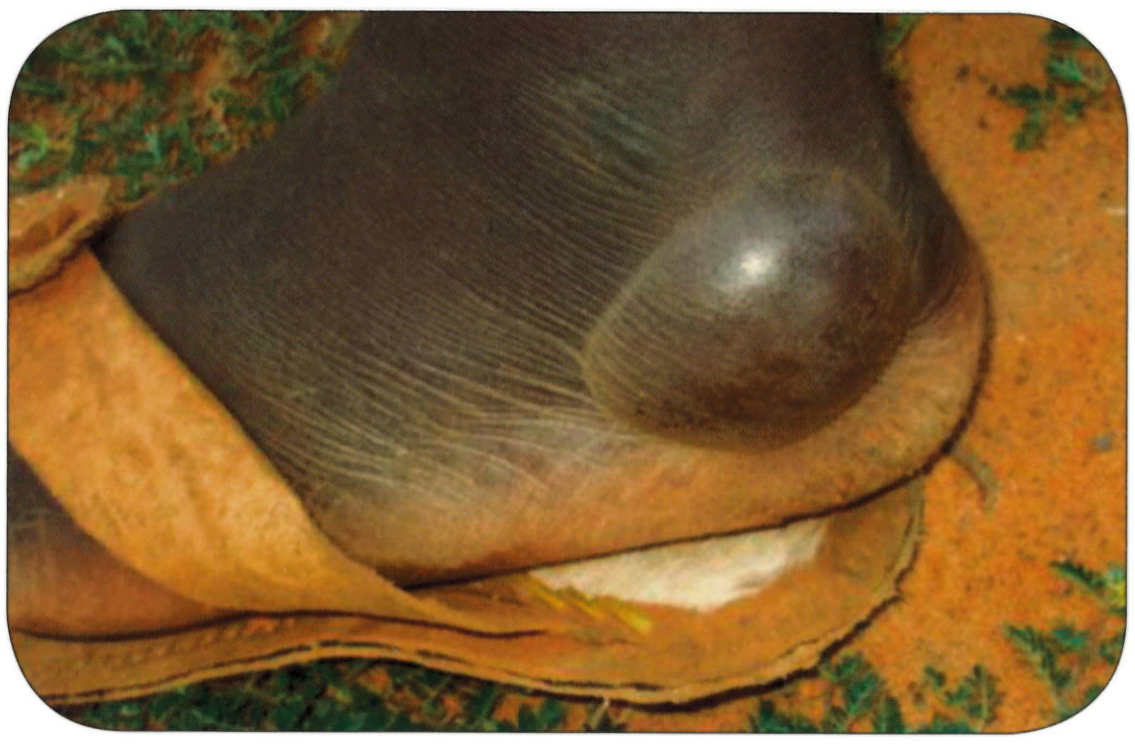

© World Health Organization, 2016

DRACUNCULIASIS

World Health Organization

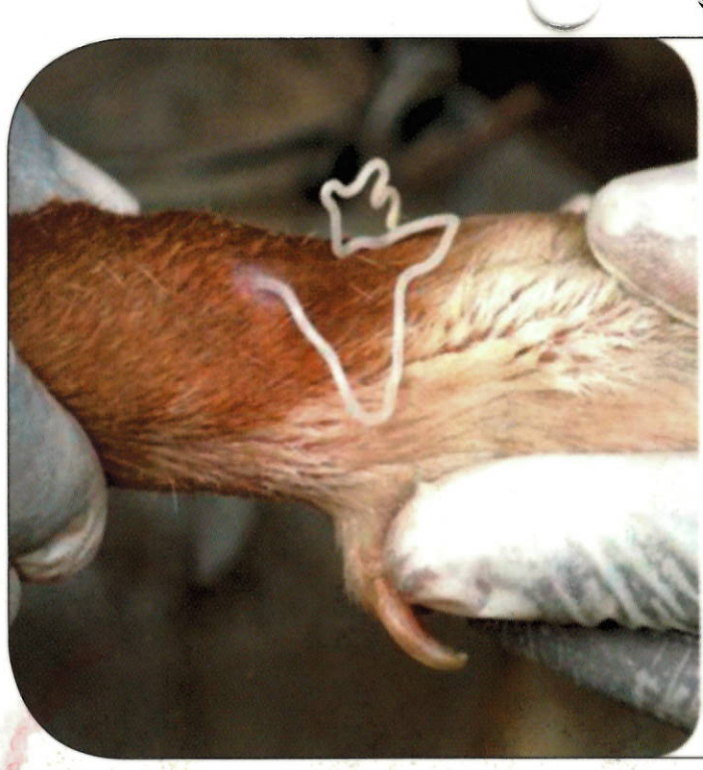

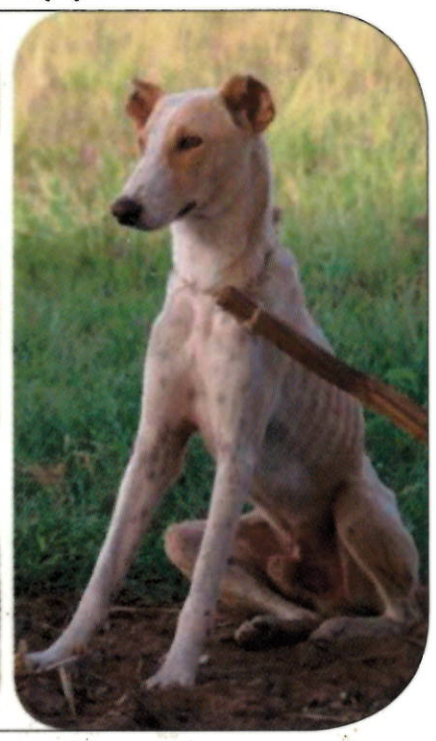

STOP GUINEA WORM DISEASE NOW
IF YOU SEE THIS, INFORM YOUR HEALTH WORKER IMMEDIATELY

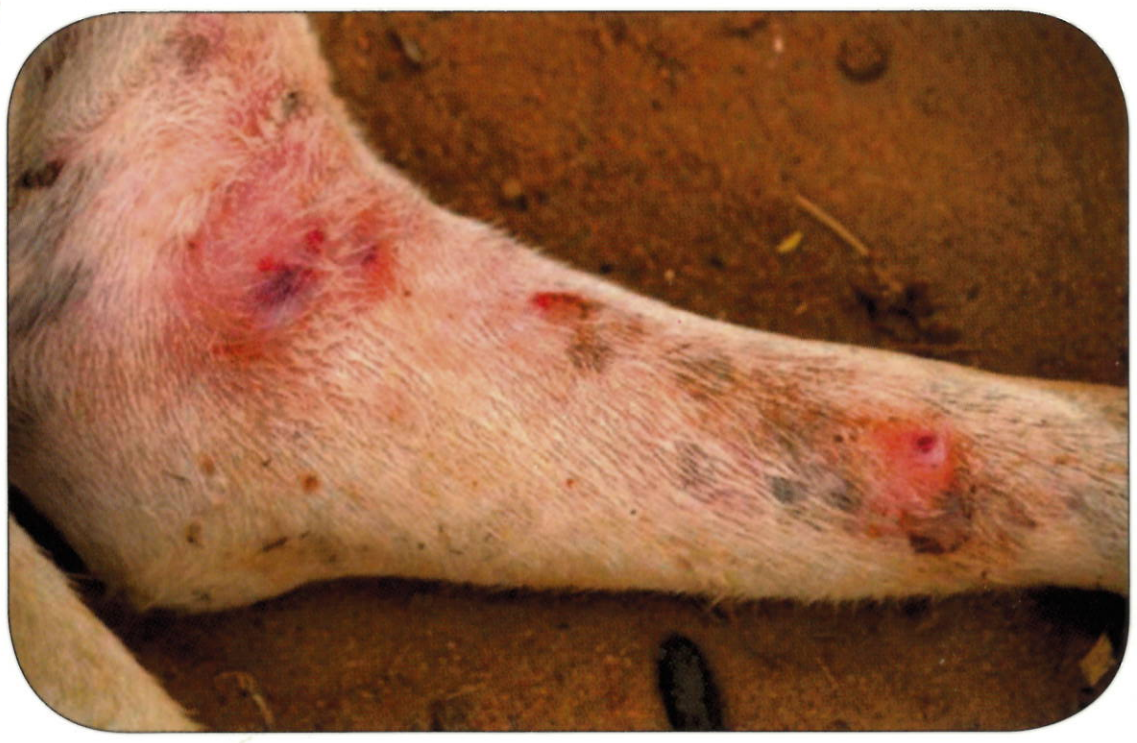

© World Health Organization, 2016
